# Recent advances in aortic valve replacement for aortic stenosis

**DOI:** 10.12688/f1000research.8728.1

**Published:** 2016-10-20

**Authors:** Ahmed Al-Adhami, Nawwar Al-Attar

**Affiliations:** 1 Department of Cardiac Surgery, Golden Jubilee National Hospital, Glasgow, G81 4DY, UK

**Keywords:** transcatheter valve, sutureless valves, Aortic valve replacement, aortic stenosis, Standard aortic valve replacement

## Abstract

Aortic valve replacement is no longer an operation that is approached solely through a median sternotomy. Recent advances in the fields of transcatheter valves have expanded the proportion of patients eligible for intervention. Comparisons between transcatheter valves and conventional surgery have shown non-inferiority of transcatheter valve implants in patients with a high or intermediate pre-operative predictive risk. With advances in our understanding of sutureless valves and their applicability to minimally invasive surgery, the invasiveness and trauma of surgery can be reduced with potential improvements in outcome. The strategy of care has radically changed over the last decade.

## Introduction

Aortic valve replacement (AVR) is the second most common cardiac procedure, and aortic stenosis (AS) is the most common valve disease. Population ageing is affecting many countries and is seen as the main driver for the increased incidence of AS in the Western world. AVR is indicated in symptomatic patients with severe stenosis (mean pressure gradient of at least 40 mmHg or maximum velocity of at least 4 m/s) or in asymptomatic patients with impaired left ventricular ejection fraction or low surgical risk ^1^. Until recently, standard AVR (SAVR) was the only curative treatment available and formed the backbone of management for most patients. With the recent publication of the Cavalier trial ^2^ of a sutureless aortic valve and the Placement of Aortic Transcatheter Valve (PARTNER) 2 randomised controlled trial ^3, 4^, an updated review of AVR is warranted. In this review, we will focus on the most important recent advances in valve technology and surgical access to the aortic valve, including sutureless and transcatheter valves and minimally invasive surgery.

## Standard aortic valve replacement

SAVR is the traditional and most common approach and is classically performed through a median sternotomy on cardiopulmonary bypass (CPB). An aortotomy is made, the diseased valve leaflets are excised, and the annulus is debrided. A biological valve, whether stented or stentless, or mechanical valve is anchored under direct vision by using interrupted or continuous sutures with or without the use of pledgets. Mechanical prostheses have excellent durability, and structural valve deterioration is extremely rare with current technology. Rates of freedom from structural valve failure in stented bioprostheses are 70 to 90% at 10 years and 50 to 80% at 15 years ^5^.

Based on extraordinary short- and long-term outcomes, it is deemed to be the gold standard operation for aortic valve disease and the benchmark against which new therapies are compared ^6^. Although improvements in the peri-operative management of critically ill elderly patients with multiple comorbidities have widened the range of patients eligible for surgery, there remains a group of patients for whom surgery would not be suitable because of excess predicted mortality.

In a recent review of 141,905 patients who underwent isolated first-time SAVR between 2002 and 2010, the vast majority of patients were in the low-risk group (80% low risk, 13.9% intermediate risk, and 6.2% high risk) ^7^. This suggests that low-risk patients still constitute the majority of patients undergoing treatment and evolving therapies targeting higher-risk groups represent a smaller proportion of the overall patient pool. Compared with the Society of Thoracic Surgeons (STS) predicted risk of operative mortality, actual in-hospital mortality experienced was significantly lower in all patients (2.5% versus 2.95%) overall and within each risk category ( *P* <0.0001). There was a notable increase in high-risk (STS score of more than 8%) and intermediate-risk (STS score of 4 to 8%) patients undergoing surgery from the earlier to the latter years, rising from 5.7 to 6.6% and 12.8 to 14.9%, respectively.

In addition to clinical fitness and prohibitive pre-operative risk, SAVR may be declined in clinically fit patients with anatomical features that pose particular intra-operative challenges such as the porcelain aorta, small aortic annulus, or previous chest radiotherapy. These shortfalls in SAVR have driven the development of alternative interventions in the form of transcatheter AVR (TAVR), sutureless AVR (SuAVR), and minimally invasive AVR (MIAVR).

## Sutureless aortic valve replacement

Sutureless aortic valves are biological pericardial prostheses designed for rapid deployment and by definition require *fewer than* four annular anchoring sutures ^8^. Currently commercially available prostheses include the Intuity Elite pericardial valve (Edwards Lifesciences, Irvine, CA, USA) and the 3F Enable (Medtronic, Minneapolis, MN, USA) and the Perceval S (Sorin, Saluggia, Italy) valves. The deployment mechanism and techniques differ between these models. The Intuity valve uses a balloon-expandable cloth-covered steel frame that is anchored to the annulus by three individual sutures. The other two valves use a self-expanding nitinol metal frame with internal ‘memory’, which allows valve deployment with (one suture, 3F enable) or without (Perceval S) the need for anchoring sutures ^8^.

## Sutureless versus standard aortic valve replacement

With only one small published randomised controlled trial demonstrating its efficacy ^9^, the evidence for SuAVR is limited mainly to comparative and single-arm observational studies ^2, 10– 13^. The major perceived advantages of sutureless valves over conventional valves are in the reduction of cross-clamp and CPB durations, both well-known risk factors for operative mortality and morbidity, and in facilitating minimally invasive surgery ^14– 16^.

## Cross-clamp times

Aortic cross-clamp times increase the risk of severe cardiovascular morbidity by 1.4% per 1-minute increase. Clinical benefits of reduced cross-clamp times are demonstrated most in patients with left ventricular ejection fraction of not more than 40% and those with diabetes ^16^. In a study of 120 patients undergoing SuAVR (n = 50) or SAVR (n = 70), Shrestha *et al*. ^17^ concluded that CPB and cross-clamp times were significantly shorter in those undergoing SuAVR (CBP: 58.7 versus 75.3 minutes; cross-clamp: 30.1 versus 58.7 minutes) with no significant difference in mortality at 5 years. Furthermore, in a meta-analysis by Phan *et al*., pooled cross-clamp and CPB times for isolated AVR from 12 observational studies were 46.5 and 56.7 minutes, respectively ^13^.

## Outcomes

In the previously mentioned meta-analysis, pooled 30-day and 1-year mortality rates after SuAVR were 2.1% and 4.9%, respectively, with acceptable incidences of stroke (1.5%), valve degeneration (0.4%), and paravalvular leak (PVL) (3.0%) ^13^. In the recently published European prospective multicentre study (the Cavalier trial) of 658 high- to medium-risk patients undergoing SuAVR using the Perceval S rapid deployment valve, Fischlein *et al*. ^2^ reported 4.5% and 3% rates of cardiac mortality and stroke at 1 year, respectively. They reported low rates of major PVL (0.6%) and endocarditis (1.4%) and no occurrences of valve thrombosis, migration, or structural valve deterioration. Despite these promising results, the longevity of the Perceval S is yet to be determined in studies of longer durations. Moreover, given that the most important effect of sutureless rapid deployment valves is in reducing ischaemic and bypass times, the greatest benefit from sutureless valves is expected to be seen in patients undergoing prolonged or combined procedures. Only 30% of patients in this study underwent combined procedures ^18^. Moreover, the Cavalier trial has been criticised because it did not adjust for the expected early learning curve when introducing such technologies and because implantations failed in 5% of patients as a result of various complications, including dislodgement, malposition, PVL, suspected aortic tear, and multiple unsuccessful attempts. These highlight the need for mandatory proctoring during the initial stages ^18^.

## Use of sutureless valves in combined and high-risk procedures

Maximal benefit from SuAVR is arguably seen in patients at higher risk of morbidity and mortality from increased operative times or, conversely, those undergoing complex or concomitant cardiac surgery with expected long CPB and cross-clamp times, to minimise operative times and their associated morbidity ^19– 22^.

Moreover, use of sutureless prostheses can be expanded to particular high-risk situations, including the presence of a porcelain aorta, calcified aortic root, and re-operative surgery, particularly in the presence of a prior aortic homograft or stentless valves ^23^. Minh *et al*. ^21^ and Vola *et al*. ^24^ have both argued for the use of SuAVR in patients with coexistent mitral disease.

## Small aortic annulus and patient-prosthesis mismatch

It is known that implantation of a relatively small-sized prosthesis in patients with a small aortic annulus may result in patient-prosthesis mismatch (PPM) with increased pressure gradients ^25^. PPM is associated with symptom persistence, reduced left ventricular mass regression, and worsened mortality ^26^.

In an observational study comparing 92 patients who underwent SuAVR (Perceval S valve) with 36 who underwent aortic root enlargement (ARE) procedures and conventional SAVR for a small aortic annulus, Beckmann *et al*. ^27^ concluded that although the SuAVR cohort underwent significantly more concomitant procedures, all operation-associated times were significantly shorter—mean operation, CPB, and cross-clamp time (147, 67, and 35 minutes, respectively)—than in ARE patients (181, 105, and 70 minutes, respectively; *P* <0.001). The mean post-operative effective orifice areas indexed to the body surface area were 0.83 ± 0.14 cm ^2^/m ^2^ following SuAVR and 0.91 ± 0.2 cm ^2^/m ^2^ following ARE ( *P* = 0.040). There was no significant difference in the rates of severe PPM (11% versus 6%) and 30-day mortality (2% versus 6%) between SuAVR and ARE, respectively.

Published recommendations state that sutureless valves should be considered the first-line treatment option for patients with several comorbidities, patients of advanced age, or patients with aortic wall abnormalities, such as a small calcified aortic root, porcelain aorta, or previously implanted aortic homograft or stentless valves ^28^.

## Minimally invasive aortic valve replacement

MIAVR is defined as an AVR procedure that is performed through a small chest wall incision as opposed to conventional full sternotomy ^29^. This approach has emerged as a comparable alternative to conventional sternotomy with the aim of reducing surgical trauma without impeding quality. It has been shown to reduce post-operative stay, post-operative pain, and blood transfusion and improve cosmesis ^30– 32^. However, one of the main obstacles to the wide adoption of MIAVR is the association with increased operative times, technical difficulty, and steep learning curves. A meta-analysis revealed a weighted mean difference of 7.9 additional minutes of cross-clamp time with MIAVR ^33^. Detractors argue that the added morbidity from increased cross-clamp and CPB times outweighs the potential benefits. Moreover, no study has yet demonstrated any survival advantage with MIAVR.

## Surgical technique

The most common method of MIAVR is via a partial upper median sternotomy which involves a 5 to 10 cm midline vertical skin incision, followed by a partial J-shaped sternotomy at the level of the third to fifth intercostal space or a V-shaped sternotomy at the level of the second or third intercostal space ^34, 35^. Another approach is via a right anterior minithoracotomy with a 5 to 7 cm skin incision made at the level of the second intercostal space, followed by ligation of the right internal thoracic artery, direct aortic cannulation, and percutaneous femoral vein cannulation with advancement of the venous cannula to the right atrium ^36^. Other described approaches include transverse sternotomy or the right parasternal approach from the second to fourth intercostal spaces.

## Sutureless valves and minimally invasive surgery

With the advent of sutureless valves, the technical difficulties attributed to anchoring conventional valves to the annulus through a limited surgical field are significantly circumvented. Borger *et al*. ^9^ published the first prospective, randomised multicentre trial comparing MIAVR using sutureless valves (Edwards Intuity valve) versus full sternotomy SAVR in 46 and 48 patients, respectively. Sutureless valve replacement was associated with significantly lower cross-clamp durations (41.3 versus 54 minutes), mean transvalvular gradients (8.5 versus 10.3 mmHg), and prevalence of PPM (0% versus 15.0%) at 3 months. Although this study was not powered to evaluate differences in mortality or morbidity, there were no clear differences in early clinical outcomes, including quality-of-life measures. Pacemaker implantation rates were higher in the sutureless cohort, but this was not statistically significant (4.3% versus 0%). This study demonstrates that MIAVR can be performed with reduced cross-clamp times if sutureless valves are used with excellent early haemodynamic performance. Combined with their lack of annular suture material, it is postulated that balloon-deployable frames allow for maximal haemodynamic prosthesis performance, as they are actively expanded into the left ventricular outflow tract ^37^. Previous non-randomised studies have also confirmed shorter procedural times, and these similarly did not translate to better outcomes with comparable in-hospital mortality and peri-operative stroke rates demonstrated ^37, 38^. Some have reported lower rates of blood transfusions, shorter intensive care unit and intubation time, and lower incidences of post-operative atrial fibrillation and respiratory insufficiency with SuAVR, which translated to significant reductions in overall cost attributed mainly to reduced overall hospital stay and diagnostics ^37^.

## Minimally invasive re-operative surgery

Minimally invasive approaches to the aortic valve have also been used in the re-operative setting. Apart from the expected technical challenges in minimal access re-operative surgery, the major concern and area of debate focus on the optimal myocardial protection strategy. Owing to the limited surgical field, it is difficult to isolate and control internal thoracic artery grafts prior to clamping ^39^. In a meta-analysis of small retrospective studies, comparisons between minimally invasive approaches to the aortic valve and conventional sternotomy in the re-operative setting did not yield any significant differences in in-hospital mortality and stroke, which ranged from 0 to 9.5% and from 2.6 to 8%, respectively ^39^.

There was no significant difference in length of hospital stay or rates of pacemaker implantation, renal failure, re-operation for bleeding, and hospital stay between the two groups. Vola *et al*. demonstrated the feasibility of using minimally invasive SuAVR in three patients with degenerated small 19 mm aortic bioprostheses with no cases of mortality and average implantation time of 10.3 minutes ^40^.

## Transcatheter aortic valve replacements

Cribier *et al*. ^41^ performed the first human TAVR in 2002. TAVR is typically targeted at patients with severe AS who are unfit for conventional surgery ^42– 45^. In our previous report ^46^, we highlighted the procedural technique and the important trials in this field. We will focus on the important advances in this field since then.

## High-risk ‘inoperable’ patient category

The use of TAVR was initially evaluated in inoperable AS. The PARTNER 1B randomised trial examined the use of SAPIEN valves versus standard treatment in 358 inoperable patients ^47^. Five-year results revealed an absolute survival benefit of 21.8% (all-cause mortality of 71.8% versus 93.6%) and a 28.4% lower cardiovascular mortality (57.5% versus 85.9%) with TAVR. Around 86% of surviving patients resided in New York Heart Association functional class 1 or 2 (no or slight limitation of physical activity). Moderate or severe PVL was present in 14% at first available measurement, and this was associated with a higher cardiovascular mortality but not all-cause mortality. No patients developed structural valve deterioration requiring re-intervention, and one patient required SAVR for endocarditis following TAVR. There was no persistent risk of stroke over 5 years beyond the early procedural risk.

The remarkable findings from this trial and subsequent trials and registries ^43, 48– 51^ formed the basis of the class I recommendation for TAVR in symptomatic inoperable severe AS in the European Society of Cardiology and American Heart Association guidelines on valve disease ^1, 52^.

## High-risk ‘operable’ category

In the PARTNER 1A trial ^53^, a randomised cohort of 699 high-risk operable patients with a mean STS predictive mortality score of 11.7% were randomly assigned to either TAVR or SAVR. TAVR was comparable to SAVR with regard to 5-year all-cause mortality (67.8% versus 62.4%; *P* = 0.76) with no instances of structural valve deterioration requiring SAVR in either group. Moderate or severe aortic regurgitation was significant higher following TAVR (14% versus 1%; *P* <0.0001) and this was associated with increased 5-year mortality (72.4% with moderate or severe aortic regurgitation versus 56.6% with mild aortic regurgitation or less; *P* = 0.003).

It is important to note that the devices used in the PARTNER 1 trials have since been superseded by second- and third-generation devices which have smaller sheaths and allow for partial repositioning and sealing of PVLs. In a randomised multicentre study of the lower-profile (smaller sheath) SAPIEN XT valve compared with the original SAPIEN valve in inoperable patients with severe, symptomatic AS ^4^, it was noted that major vascular and bleeding complications were significantly reduced with no detrimental effect on the primary outcomes of all-cause mortality, major stroke, or rehospitalisation. Both overall and major vascular complications were higher at 30 days in patients undergoing TAVR with SAPIEN compared with SAPIEN XT (overall: 22.1% versus 15.5%; major: 15.2% versus 9.5%; *P* = 0.04). Questions relating to long-term durability of transcatheter valves beyond 5 years remain to be definitively answered.

## Intermediate-risk category

Following encouraging results in high-risk cohorts, there has been an increasing trend in the use of transcatheter valves in intermediate- and low-risk patient profiles. In a recent trial ^3^ evaluating the use of a second-generation SAPIEN XT system in ‘intermediate’-risk patients with severe AS, 2,032 patients entered two parallel prospective, randomised multicentre trials comparing TAVR and SAVR (PARTNER 2). Although these patients are lower risk than those included in previous trials, they still relate to a relatively high-risk population (STS risk score of at least 4.0% and not more than 8.0%) ^52^. TAVR was comparable to surgery at 2 years in terms of combined all-cause mortality or disabling stroke (19.3% versus 21.1%; *P* = 0.33), all-cause mortality (16.7% versus 18%; *P* = 0.45), and the rate of disabling stroke (6.2% versus 6.4%; *P* = 0.83). In the transfemoral-access TAVR cohort, there were lower rates of death or disabling stroke than in the surgery cohort, and in the transthoracic-access cohort outcomes were similar to surgery. Major vascular complications were more frequent in the TAVR group than in the surgery group (7.9% versus 5.0%; *P* = 0.008). TAVR was associated with larger post-operative aortic valve areas (1.5 ± 0.4 versus 1.4 ± 0.4 cm; *P* <0.001) and lower rates of renal injury, life-threatening bleeding, and new-onset atrial fibrillation than in the surgery group. The rates of new pacemaker implantations were similar at 2 years (TAVR 11.8% versus SAVR 10.3%; *P* = 0.29). The surgical cohort experienced a lower rate of moderate or severe PVL at 2 years (0.6% versus 8% after TAVR; *P* = 0.43). In the TAVR cohort, moderate or severe PVL at 30 days conferred a higher mortality at 2 years than trace or no regurgitation ( *P* <0.001). The risks of all major complications with TAVR were lower than in earlier randomised trials.

## Young patient category

No published randomised trials have evaluated the use of TAVR against SAVR in young cohorts. Extrapolating available data from completed high- and intermediate-risk studies is problematic because of several important limitations. Firstly, the mean age of patients recruited in all major randomised trials ^3, 45, 53^ exceeded 75 years; the majority had a mean age of over 80 years. Furthermore, as younger, more active patients are more likely to live longer and be affected by structural valve deterioration, the lack of long-term durability data for TAVR is a particular concern. Moreover, the physiological impact of PVLs after TAVR, a known predictor of mortality in older patients, is yet to be established in younger subjects, and appropriately designed studies are required ^54^.

## Sutureless versus transcatheter aortic valve replacement

The advantages of SuAVR over TAVR include its facilitation of direct valve visualisation (versus fluoroscopic guidance) and sizing and its valve excision and annular decalcification, which may reduce the incidence of PVL and cerebral and systemic embolisation of calcium debris. Santarpino *et al*. ^55^ compared 37 propensity-matched pairs of patients who underwent SuAVR (Perceval) and TAVR (SAPIEN and SAPIEN XT). Although there was no statistically significant difference (likely secondary to the small sample size), there was a trend towards higher mortality in the TAVR group (8.1% versus 0%; *P* = 0.24) and a higher pacemaker implantation rate in the sutureless group (10.8% versus 2.7%; *P* = 0.18). Higher pre-discharge PVL rates (13.5% versus 0%; *P* = 0.027) and lower accrual survival (86.5% versus 97.3%) at mean follow-up of 18.9 months were seen following TAVR compared with SuAVR. Muneretto *et al*. ^56^ compared 53 and 55 patients with an intermediate- to high-risk profile (STS score of more than 4%) who underwent SuAVR (Perceval) and TAVR (CoreValve), respectively. The TAVR group was associated with higher pacemaker implantations (25.5% versus 2%) and peripheral vascular complications (14.5% versus 0%) than SuAVR, but there was no difference in in-hospital mortality or overall survival when compared with SAVR.

## The Heart Team

It is important to note that a comprehensive assessment by a multidisciplinary ‘Heart Team’ is essential for all patients being considered for transcatheter valves, sutureless valves, MIAVR, and other emerging technologies and procedures. The Heart Team, consisting of cardiac surgeons, interventional cardiologists, anaesthesiologists, and specialists in cardiac imaging, among others, optimise patient selection through assessment of clinical fitness, evaluation of the risk/benefit ratio of different therapeutic strategies, assessment of access suitability and valvular and root anatomy, and appraisal of local experience and outcomes prior to arriving at a consensus on the optimal treatment ^57^.

## Upcoming trials

It is important that all interpretations and extrapolations made from the data in the above studies take into account the rapidly developing technological field. Many devices used in these studies have been superseded by newer valves. In the case of TAVR, newer lower-profile delivery systems are expected to lower potential vascular access complications and widen the eligibility for transfemoral TAVR that is known to have better outcomes.

Another randomised trial evaluating the ‘intermediate’-risk population using the transcatheter Medtronic CoreValve against SAVR—Symptomatic Aortic Stenosis in Intermediate Risk Subjects Who Need Aortic Valve Replacement (SURTAVI) trial ^58^—has completed recruitment, and follow-up data are awaited. Two large randomised trials ^59, 60^ evaluating the use of TAVR in ‘low’-risk patients are currently recruiting. Moreover, the United Kingdom Transcatheter Aortic Valve Implantation (UK TAVI) trial is a randomised trial of any commercially available device and includes newer-generation devices. With regard to sutureless valves, the first international, prospective, post-market randomised multicentre trial of Perceval Sutureless valves versus SAVR is under way: Perceval Sutureless Implant versus Standard AVR (PERSIST-AVR) trial ^61^.

## Conclusions

Although multicentre registry and randomised data have suggested non-inferiority of TAVR in high- and intermediate-risk populations when compared with SAVR, the mean age of patients recruited in comparative trials exceeded 75 years; hence, extrapolation of trial results to younger age groups is problematic, particularly given the lack of long-term durability data. SAVR should therefore remain the treatment of choice in all patients below 75 years of age until appropriate trial data become available and long-term durability is established. For patients with a high operative risk or those older than 75 years, decisions regarding the most appropriate treatment strategy should be undertaken by the Heart Team following a careful review of the risks and benefits of each approach.

Midterm durability data for sutureless valves are now available, but, as with TAVR, there is a paucity of long-term durability data in contrast to conventional stented bioprostheses and mechanical valves. By allowing direct valve visualisation and sizing, valve excision and annular debridement, shorter cross-clamp times, simpler and faster atraumatic implantation with minimal valve crimpling, and avoidance of annular anchoring sutures, SuAVR addresses the limitations of both TAVR and SAVR and hence offers a promising alternative that may also serve as a first-line treatment for patients lying in the grey zone between surgery and TAVR. Short-term clinical data indicate mortality and morbidity rates similar to those of SAVR, with a satisfactory haemodynamic profile. This evidence, however, is based mainly on underpowered non-randomised data, and although a multicentre randomised trial is under way, international retrospective and prospective registry data are eagerly needed to allow for future well-powered propensity score-matched analyses of the durability and long-term complication profile of SuAVR. By reducing operative times and simplifying the surgical technique, SuAVR may provide the necessary impetus for the adoption of MIAVR worldwide. Future trials should compare minimally invasive SuAVR to SAVR in the setting of high- and intermediate-risk patient profiles.

## Abbreviations

ARE, aortic root enlargement; AS, aortic stenosis; AVR, aortic valve replacement; CPB, cardiopulmonary bypass; MIAVR, minimally invasive aortic valve replacement; PARTNER, Placement of Aortic Transcatheter Valve (trial); PPM, patient-prosthesis mismatch; PVL, paravalvular leak; SAVR, standard aortic valve replacement; SuAVR, sutureless aortic valve replacement; STS, Society of Thoracic Surgeons; TAVR; transcatheter aortic valve replacement.
